# Dementia and Related Comorbidities in the Population Aged 90 and Over in the Vitality 90+ Study, Finland: Patterns and Trends From 2001 to 2018

**DOI:** 10.1177/08982643221123451

**Published:** 2022-10-18

**Authors:** Pauliina Halonen, Linda Enroth, Esa Jämsen, Saritha Vargese, Marja Jylhä

**Affiliations:** 1 Faculty of Social Sciences (Health Sciences), 7840 Tampere University, Tampere, Finland; 2 Gerontology Research Center, Tampere, Finland; 3 Faculty of Medicine and Health Technology, 7840 Tampere University, Tampere, Finland; 4 Department of Geriatrics, 7840 Tampere University HospitalHospital, Tampere, Finland

**Keywords:** dementia, comorbidity, oldest old, time trends

## Abstract

**Objectives:**

To examine trends in the prevalence of dementia and related comorbidities among the oldest old.

**Methods:**

Six repeated cross-sectional surveys were conducted between 2001 and 2018, each including all inhabitants aged over 90 in Tampere, Finland (*n* = 5386). Co-occurring conditions and their time trends among participants with dementia were examined using logistic regression and generalized estimating equations.

**Results:**

The prevalence of dementia decreased from 47% in 2007 to 41% in 2018. Throughout the study period, depression was more common among people with dementia compared to those without. The prevalence of hypertension, diabetes, and osteoarthritis increased and the prevalence of depression decreased among people with dementia. The mean number of comorbidities increased from 2.0 in 2001 to 2.3 in 2018.

**Discussion:**

Dementia remains highly prevalent among the oldest old and it is accompanied by an increasing burden of comorbidities, posing a challenge to people with dementia, their caregivers, and care systems.

## Introduction

Dementia is associated with increased mortality, disability, lower quality of life (Andersen et al., 2004; Doblhammer & Barth, 2018; Halonen et al., 2019; Tonelli et al., 2017), and long-term care use (Forma et al., 2011; Halonen et al., 2019). It is a highly age-related condition: the incidence of dementia is highest in people aged over 90 years (Lucca et al., 2020; Olfson et al., 2021). Several recent studies imply that the incidence and prevalence of dementia are decreasing (Gao et al., 2019; Sullivan et al., 2019), but trends among the oldest old have received less research attention.

Chronic conditions rarely occur alone in old age, and dementia too is usually accompanied by other conditions. In research and clinical practice, the term comorbidity is used to refer to additional conditions coexisting with an index disease (Feinstein, 1970; Nicholson et al., 2019), while multimorbidity is defined as co-occurring diseases with no special interest on any single condition (Nicholson et al., 2019). Frequent comorbidities in dementia include hypertension, coronary heart disease, and other cardiovascular diseases, cerebrovascular diseases, diabetes, connective tissue disease (Clague et al., 2016; Jørgensen et al., 2018; Nelis et al., 2018; Wang et al., 2018), and depression (Nelis et al., 2018; Wang et al., 2018). Earlier studies using different samples and designs have found approximately as many (Schubert et al., 2006; Zekry et al., 2008), less (Forma et al., 2011; Jørgensen et al., 2018; Sherzai et al., 2016), and more (Clague et al., 2016; Wang et al., 2018) comorbidities among people with dementia than among those without.

In studies examining clusters or patterns of comorbidity, dementia is often associated with neuropsychiatric or psychogeriatric disorders (Nguyen et al., 2018; Prados-Torres et al., 2012; Schäfer et al., 2010). A history of multimorbidity has also been found to predict dementia. Grande et al. (2021) showed that individuals with neuropsychiatric and cardiovascular multimorbidity and those with sensory impairment or cancer were at increased risk for dementia, but no association was found between the patterns of respiratory, metabolic, and musculoskeletal conditions and dementia development. With the exception of Sherzai et al. (2016), earlier studies have been conducted in samples combining age groups of people aged over 65 years, either in care settings or using insurance registers. A recent Finnish study based on national population register data showed that among people who died at the age of 70 or over, the proportion of those with a dementia diagnosis during the last 5 years of life increased from 24.5% in 2001 to 35.6% in 2013, and within this group, the number of comorbidities increased. An increasing trend was seen for hypertension, cardiac insufficiency, osteoporosis, insomnia, diabetes, cancer, lipoprotein disorders, renal insufficiency, and thyroid disorders. These growth trends are likely due to increasing age at death, longer survival with chronic diseases, and improving diagnostic practices (Vargese et al., 2021).

This study focuses on persons aged over 90, who show the highest prevalence and incidence of dementia. This population segment has not yet been extensively researched, mainly because it has only emerged quite recently as a major population group, but also because of the challenges involved in studying individuals with multiple health problems and the rather large numbers living in residential care (Jylhä et al., 2020). The rapid absolute and relative growth of this population segment worldwide nonetheless makes it one of great interest and importance. In Finland, the number of people aged 90 and over is projected to rise from around 23,000 (.4% of the total population) in 2000 to over 130,000 (2.3%) by 2040 (Official Statistics of Finland, 2021). In the US, the size of this age group is expected to quadruple from 2000 to 2040 and its proportion of the total population to grow from .5% to 1.6% (United Nations, Department of Economic and Social Affairs, Population Division, 2019). Prior research shows that the morbidity profile of the oldest old differs from that of the younger olds, showing high rates of dementia, cerebrovascular diseases, arthritis, and diabetes (Doblhammer & Barth, 2018; Salminen et al., 2012). Furthermore, it is not clear to what extent the trend of increasing morbidity in older people is seen in the oldest old people with dementia.

This study uses six repeated population-based cross-sectional surveys with the exact same methods to investigate the trend in the prevalence of dementia, and the patterns of related comorbidity among people aged 90 and older. We analyzed (1) the prevalence of dementia, (2) the number of chronic conditions among people with and without dementia, and (3) the prevalence of the most common comorbidities among people with dementia between 2001 and 2018.

## Methods

### Data

The data came from the Vitality 90+ Study, a population-based survey in Tampere, Finland (2019 population 238,140, of whom .9% were over 90 years). Tampere is Finland’s third-largest city, located in the Pirkanmaa region where life expectancy is close to national average (81.85 vs 81.46 in 2018) (Official Statistics of Finland, 2018). Mailed questionnaires were sent to all inhabitants aged 90 or over, irrespective of health or place of living in Tampere in 2001, 2003, 2007, 2010, 2014, and 2018. The response rates were 84%, 86%, 82%, 80%, 80%, and 77%, respectively. Proxy respondents were used in order to obtain information from individuals with cognitive problems or other health issues and so to make the study more representative. Participants were categorized as self-respondents if they chose the response options themselves (even if they received help with writing from a family member, relative or acquaintance, or home care worker/nursing staff). Respondents were considered proxy if a family member, relative or acquaintance, or home care worker/nursing staff answered on behalf of the participant.

In each survey year, the questionnaire included an item about chronic conditions: “Has a doctor told you that you have…?”. Dementia was considered to be present in participants who answered “yes” to the question about having “dementia, Alzheimer’s disease, or worsening of memory.” Once a participant had reported dementia that was assumed to apply for the next study rounds as well because of the chronic nature of dementia. Altogether 136 (2.5%) participants reported no dementia in at least one round of data collection after reporting it in a previous round.

In addition to dementia, the presence of hypertension (high blood pressure), heart disease (coronary artery disease, arrhythmia, or myocardial infarction), stroke, diabetes, arthritis, hip fracture, and depression (depressed mood) was asked every study year. Cancer was not included in 2010 and Parkinson’s disease was not included in 2018. Chronic lung disease was listed only in the latest survey in 2018. These conditions were excluded from trend analysis.

The Vitality 90+ Study has obtained ethical permission for every survey round from the regional ethics committee of Tampere University Hospital (in 2018 approval number R18041) or the City of Tampere, depending on the year of the survey. Written informed consent was obtained from the participants or their representative.

### Statistical Analysis

The data were first analyzed cross-sectionally separately in each survey year to define the frequency of each condition. In order to identify the most common combinations of chronic conditions among participants with dementia, we determined the prevalence of pairs of conditions among them. We then formed triads of conditions that belonged to the pairs that had a prevalence higher than 10% and further analyzed the most common pairs and triads among participants with dementia (Supplement Table 1). Logistic regression analysis was performed for each survey year, with dementia as the dependent variable. The analysis was done first with individual chronic conditions and the number of conditions (multimorbidity), and then with pairs and triads of chronic conditions as independent variables.

Trend analysis was then performed to examine linear trends in the prevalence of chronic conditions over time with a generalized estimating equation (GEE). With this approach, it is possible to model the changes in the population mean over time with repeated cross-sectional measurements, representing the population at each time point. Since all individuals aged over 90 in the area were included in the study at all six time points, two-thirds (69%) of the participants responded in one survey round, 25% in two rounds, and 6% in three or more rounds. An independent ‘working’ correlation structure was used to account for repeated responses by the same individuals across several study years (Raitanen et al., 2020). A binomial distribution family with logit link function, reporting odds ratios (ORs) with 95% confidence intervals (CIs), was used to analyze the trend in the prevalence of chronic conditions. A negative binomial distribution family with log link function, reporting incidence rate ratio (IRR) with CIs, was used to analyze the trend in the number of chronic conditions over time. In this latter model, the number of chronic conditions was used as a continuous variable. All models included study year as the independent variable and were first run without adjustments and then adjusted for age and gender. With no major differences between the unadjusted and adjusted models, only the age- and gender-adjusted models are presented. The figures show fitted lines derived from the age- and gender-adjusted models and the observed prevalence for each study year, with a *p*-value for the differences between the years. A *p*-value of .05 or lower was considered statistically significant. The analysis was performed with Stata 15 (StataCorp LLC).

## Results

### Characteristics of the Study Population and the Prevalence of Dementia

**Figure 1.**
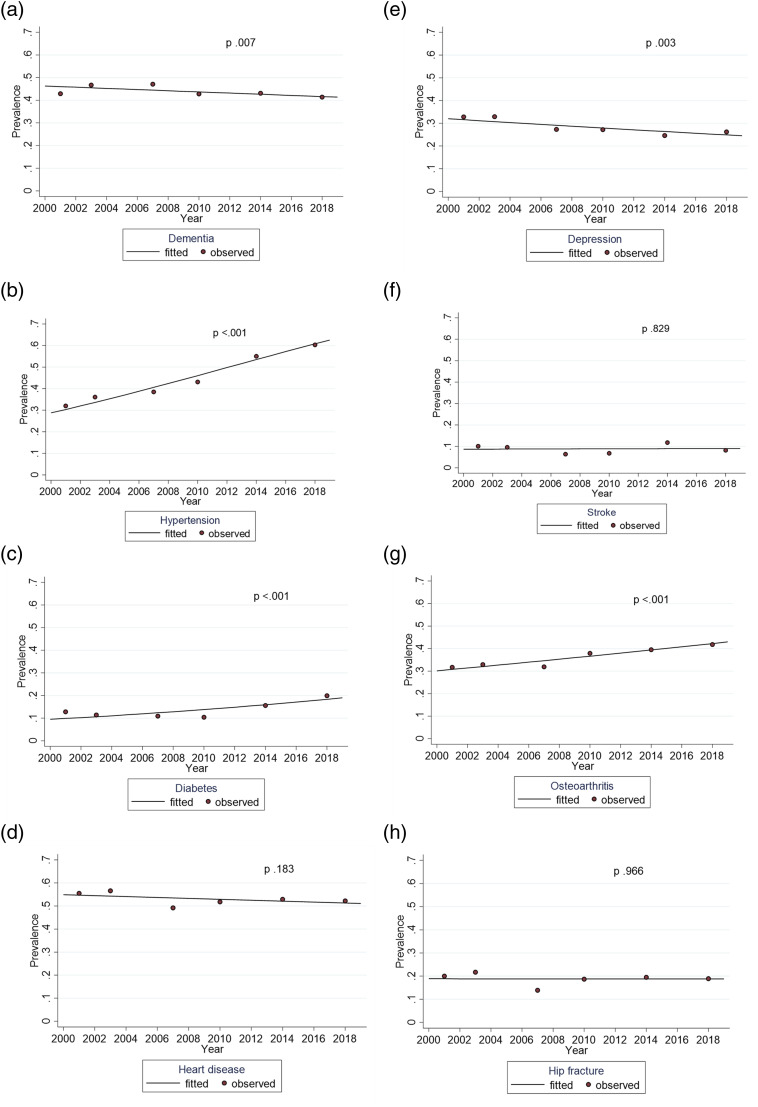
Trends for dementia and comorbid chronic conditions among participants with dementia between 2001 and 2018, adjusted for age and gender.

After the removal of 105 observations with missing information on dementia, 7483 observations and 5386 participants were included in the analysis. Most of the participants were women, but the proportion of men increased over the years. The study population grew over the years from 874 participants to 1856 participants. The prevalence of dementia was 43% in 2001, 47% in 2003, 47% in 2007, 43% in 2010, 43% 2014, and 41% in 2018, showing a decreasing trend from 2007 onwards (*p* .007) (Table 1 & Figure 1(a)). The absolute number of participants with dementia increased despite the proportional decrease. Participants with dementia were slightly older than those without dementia. No gender differences were found in dementia prevalence in any survey year. People with dementia lived in long-term care more often than those without dementia.

**Table 1.** Characteristics of the Study Population With and Without Dementia From 2001 to 2018 in the Vitality 90+ Study.

	2001		2003		2007		2010		2014		2018		
	(*n* = 874)		(*n* = 937)		(*n* = 932)		(*n* = 1263)		(*n* = 1621)		(*n* = 1856)		
	Dementia *n* (%)	No dementia *n* (%)	Dementia *n* (%)	No dementia *n* (%)	Dementia *n* (%)	No dementia *n* (%)	Dementia *n* (%)	No dementia *n* (%)	Dementia *n* (%)	No dementia *n* (%)	Dementia *n* (%)		No dementia *n* (%)
	375 (42.9)	499 (57.1)	438 (46.7)	499 (53.3)	439 (47.1)	493 (52.9)	541 (42.8)	722 (57.2)	698 (43.1)	923 (56.9)	768 (41.4)		1088 (58.6)
Age (years), mean	92.5	92.2*	92.8	92.1***	92.9	92.3**	93.1	92.2***	93.0	92.3***	93.0		92.5***
Range	90–106	90–104	90–103	90–106	90–105	90–104	90–107	90–107	90–106	90–103	90–105		90–107
Gender													
Women	309 (82.4)	395 (79.2)	360 (82.2)	396 (79.4)	351 (80.0)	392 (79.5)	577 (79.9)	449 (83.0)	542 (77.7)	707 (76.6)	575 (74.9)		793 (72.9)
Men	66 (17.6)	104 (20.8)	78 (17.8)	103 (20.6)	88 (20.1)	101 (20.5)	92 (17.0)	145 (20.1)	156 (22.4)	216 (23.4)	193 (25.1)		295 (27.1)	
Proxy	158 (42.1)	47 (9.5) ***	154 (35.2)	53 (10.6) ***	130 (29.8)	12 (2.4) ***	238 (44.2)	41 (5.7) ***	270 (39.2)	39 (4.3) ***	251 (32.7)		34 (3.1) ***	
In long-term care	223 (59.5)	116 (23.3) ***	226 (51.6)	116 (23.3) ***	238 (54.3)	83 (16.9) ***	327 (60.9)	142 (19.8) ***	404 (58.2)	164 (18.0)***	408 (53.5)		143 (13.2) ***	
Chronic conditions	
Hypertension	120 (32.0)	158 (31.7)	158 (36.1)	200 (40.1)	169 (38.5)	253 (51.3) ***	233 (43.1)	427 (59.1) ***	384 (55.0)	606 (65.7) ***	463 (60.3)		731 (67.2)**
Heart disease	208 (55.5)	259 (51.9)	248 (56.6)	270 (54.1)	216 (49.2)	275 (55.8) **	280 (51.8)	409 (56.7)	369 (52.9)	499 (54.1)	401 (52.2)		565 (51.9)	
Cancer	47 (12.5)	49 (9.8)	52 (11.9)	62 (12.4)	61 (13.9)	62 (12.6)			110 (15.8)	155 (16.8)	110 (14.3)		225 (20.7) ***	
Stroke	38 (10.1)	31 (6.2)*	42 (9.6)	29 (5.8)*	28 (6.4)	24 (4.9)	37 (6.8)	30 (4.2) *	82 (11.8)	63 (6.8) **	63 (8.2)		67 (6.2)	
Diabetes	48 (12.8)	47 (9.4)	50 (11.4)	45 (9.0)	48 (10.9)	55 (11.2)	56 (10.4)	93 (12.9)	109 (15.6)	141 (15.3)	153 (19.9)		194 (17.8)	
Osteoarthritis	119 (31.7)	194 (38.9)*	144 (32.9)	177 (35.5)	140 (31.9)	229 (46.5) ***	205 (37.9)	339 (47.0) **	276 (39.5)	434 (47.0) **	321 (41.8)		505 (46.4)*	
Chronic lung disease											64 (8.3)		89 (8.2)	
Parkinson’s disease	14 (3.7)	7 (1.4)*	12 (2.7)	6 (1.2)	11 (2.5)	5 (1.0)	8 (1.5)	10 (1.4)	18 (2.6)	6 (.7) **		
Hip fracture	75 (20.0)	77 (15.4)	95 (21.7)	78 (15.6)*	61 (13.9)	105 (21.3) **	101 (18.7)	116 (16.1)	136 (19.5)	142 (15.4)*	145 (18.9)		128 (11.8) ***
Depression	123 (32.8)	85 (17.0) ***	144 (32.9)	82 (16.4) ***	120 (27.3)	82 (16.6) ***	147 (27.2)	93 (12.9) ***	172 (24.6)	109 (11.8) ***	201 (26.2)	107 (9.8) ***
No. of chronic conditions (0–7)													
Mean	2.0	1.7**	2.0	1.8**	1.8	2.1***	2.0	2.1*	2.2	2.2	2.3		2.1*	
SD	1.2	1.2	1.3	1.2	1.2	1.3	1.3	1.2	1.3	1.2	1.4		1.2

*p*-value for chi-square test (categorical variables) and Mann–Whitney-U test (age and no. of chronic conditions).

Notes: Dementia not included in the number of conditions.

SD = Standard deviation.

*p < .05; **p < .01; ***p < .001.

The use of a proxy respondent was considerably more common among participants with dementia compared to participants without dementia. Around 30%–40% of participants reporting dementia had answers provided by a proxy respondent, while among participants without dementia proxy respondents were rare: between 2% and 10% depending on the survey year (Table 1). Proxies were most often relatives, but survey answers were also provided by staff in nursing homes.

### Chronic Conditions Among Participants with and without Dementia

The mean number of chronic conditions was in most survey years higher for participants with dementia compared to those without. In all survey years the most common chronic conditions among participants with dementia were heart disease and hypertension, followed by osteoarthritis and depression (Table 1). In most survey years, depression, stroke, Parkinson’s disease, and hip fracture were more prevalent among participants with dementia than those without. In contrast, osteoarthritis and hypertension were more prevalent among those without dementia (Table 1).

Hypertension, heart disease, and osteoarthritis formed the most prevalent combinations of two and three conditions among participants with dementia (Supplement Table 1).

Age- and gender-adjusted logistic regression showed that the odds of depression and Parkinson’s disease were more than twice as high among participants with dementia than among those without. In most survey years the odds of having a stroke and a hip fracture with dementia were above 1.00 compared with participants without dementia, but not always statistically significant. The odds of hypertension and osteoarthritis, on the other hand, were lower for participants with dementia compared to those without dementia. Participants with and without dementia did not differ in the odds of having heart disease, diabetes, or cancer. The association of the number of other morbidities with dementia fluctuated from one study year to another but was positive in the beginning and at the end of the study period (Table 2).

**Table 2.** Association of Chronic Conditions and Multimorbidity With Dementia in the Six Study Rounds. Logistic Regression Analysis With Odds Ratios (OR) and 95% Confidence Intervals (CI) Adjusted for Age and Gender.

	2001		2003		2007		2010		2014		2018	
	OR	95% CI	OR	95% CI	OR	95% CI	OR	95% CI	OR	95% CI	OR	95% CI
**Comorbid condition**												
Hypertension	1.00	0.75–1.34	.84	.64–1.11	**.61**	**.47–.79**	**.54**	**.43–.68**	**.64**	**.52–.78**	**.74**	**.61–.89**
Heart disease	1.15	0.88–1.50	1.12	.86–1.46	**.76**	**.59–.99**	.83	.66–1.05	.95	.78–1.15	1.00	.83–1.20
Cancer	1.36	.89–2.08	.94	.63–1.41	1.13	.77–1.67	—	—	.93	.71–1.22	**.64**	**.50–.82**
Stroke	**1.73**	**1.06–2.85**	**1.82**	**1.11–2.98**	1.34	.76–2.36	**1.67**	**1.01–2.76**	**1.85**	**1.31–2.62**	1.40	.98–2.01
Diabetes	1.44	.94–2.21	1.32	.86–2.03	.99	.64–1.46	.83	.58–1.19	1.11	.84–1.46	1.20	.94–1.52
Osteoarthritis	**.72**	**.54–.96**	.90	.68–1.18	**.54**	**.41–.71**	**.66**	**.53–.84**	**.74**	**.61–.91**	**.79**	**.65–.96**
Chronic lung disease	—	—	—	—	—	—	—	—	—	—	1.04	.74–1.46
Parkinson’s disease	**2.64**	**1.05–6.62**	2.25	.83–6.12	2.74	.94–8.00	1.01	.39–2.61	**4.19**	**1.64–10.67**	—	—
Hip fracture	1.31	.92–1.86	**1.42**	**1.02–1.99**	**.56**	**.39–.79**	1.11	.82–1.50	**1.31**	**1.01–1.70**	**1.69**	**1.30–2.19**
Depression	**2.42**	**1.76–3.34**	**2.50**	**1.83–3.41**	**2.05**	**1.48–2.83**	**2.54**	**1.89–3.40**	**2.54**	**1.94–3.32**	**3.25**	**2.51–4.20**
**No. of comorbidities**												
	**1.18**	**1.06–1.33**	**1.18**	**1.06–1.31**	**.83**	**.75–.93**	.92	.84–1.01	1.03	.95–1.11	**1.10**	**1.02–1.19**

Notes: Analyses were conducted for each condition and the number of comorbidities separately.

No. of comorbidities range between 0 and 7.

Bolding indicates a statistically significant association.

The results of the regression models for combinations (pairs and triads) of comorbid conditions were closely similar to those of single conditions comorbid with dementia. The pair of hypertension and osteoarthritis was less likely to co-occur with dementia throughout the survey years, with odds ranging from .44 (CI 0.32–.62) in 2007 to .74 (CI 0.60–.91) in 2018. Pairs and triads including depression showed higher odds to occur with dementia in later study years (Supplement Table 2).

### Trends for Comorbidities of Dementia

**Figure 2.**
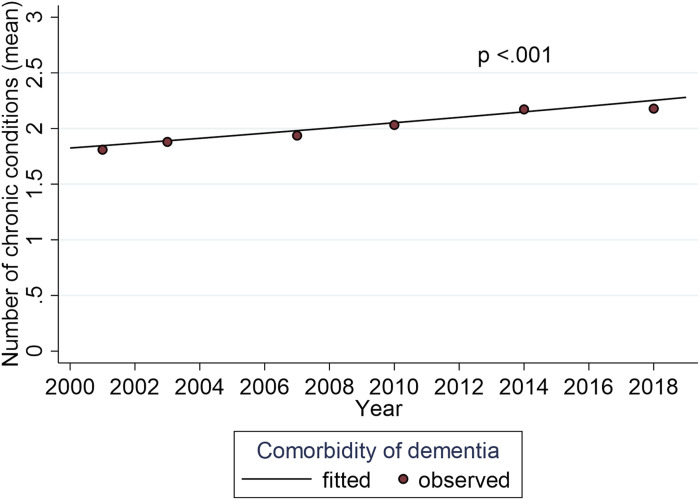
Trend for number of comorbidities with dementia between 2001 and 2018, adjusted for age and gender.

Figure 1 shows the trends for dementia and conditions comorbid with dementia from GEE models (fitted lines). The adjusted prevalence of hypertension and diabetes comorbid with dementia nearly doubled during the study period (*p* <.001) and that of osteoarthritis also increased markedly (*p* <.001), particularly in later survey years. The prevalence of heart disease, stroke, and hip fracture comorbid with dementia was steady throughout the study years (*p* .183, *p* .829, *p* .966, respectively). The only condition showing a decreasing trend over time was depression (*p* .003) (Figure 1). The mean number of conditions comorbid with dementia increased from 2.0 to 2.3 during the study period (*p* <.001) (Figure 2). It is of note that despite the stable or decreasing proportions, the absolute number of people with heart disease, hip fracture, and depression increased throughout the study years due to the increasing size of the basic population (Table 1).

**Figure 3.**
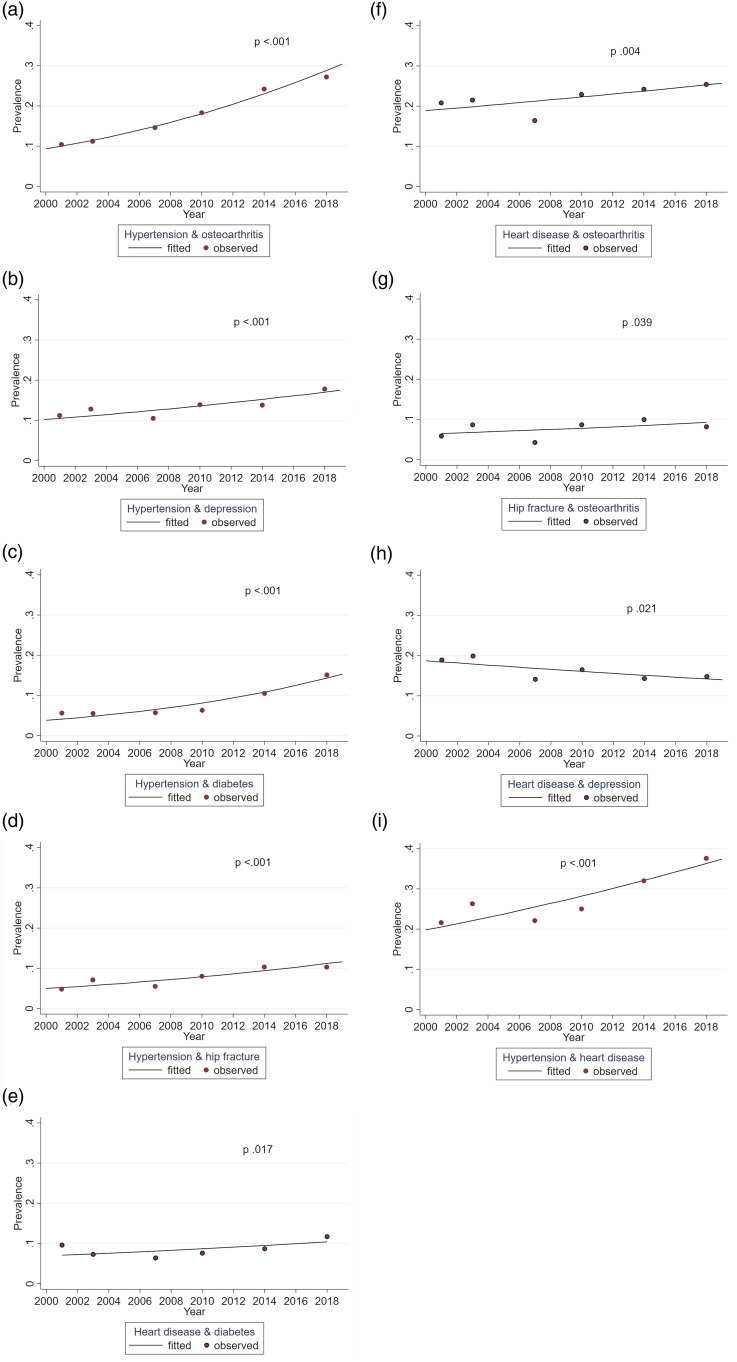
Trends for pairs of comorbid chronic conditions among participants with dementia between 2001 and 2018, adjusted for age and gender.

**Figure 4.**
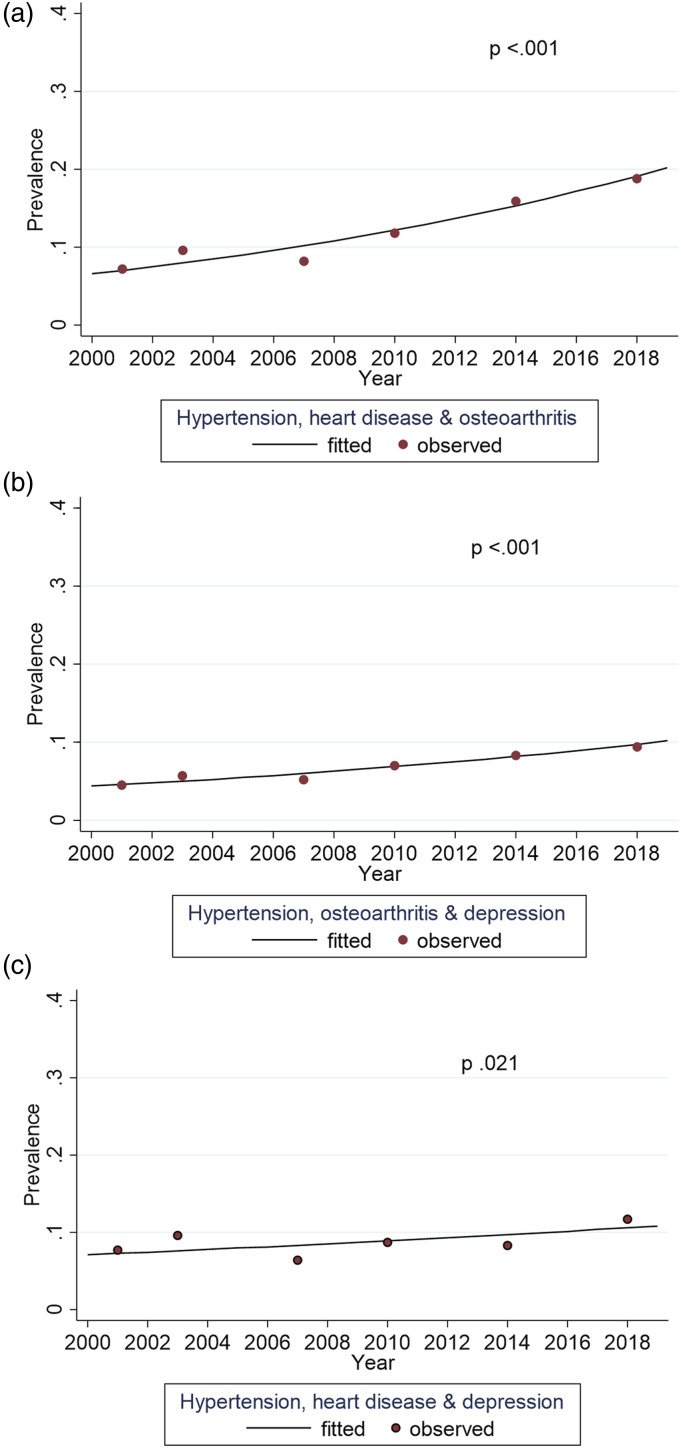
Trends for triads of comorbid chronic conditions among participants with dementia between 2001 and 2018, adjusted for age and gender.

Figure 3 and Figure 4 present combinations of conditions comorbid with dementia that demonstrated a significant trend over the study years. Driven by the large increase in hypertension, all combinations including hypertension became more prevalent over time. The largest increases were seen for a combination of hypertension and osteoarthritis (*p* <.001) and hypertension and diabetes (*p* <.001). The only combination with a decreasing trend was heart disease and depression (*p* .021).

## Discussion

This study examined time trends of dementia and its comorbidities in people aged 90 and over during a 17-year period. Parkinson’s disease, depression, hip fracture, and stroke were more likely to co-occur with dementia than to occur without it, while hypertension and osteoarthritis were more prevalent among those without dementia. In particular, the prevalence of hypertension, diabetes, and osteoarthritis increased in time, whereas the prevalence of hip fracture, stroke, and heart disease remained stable. The only condition showing a decreasing trend was depression. The number of chronic conditions comorbid with dementia increased over the study period. To our knowledge, this is the first population-based study examining time trends in dementia comorbidity in the oldest old population.

The prevalence of dementia was higher than 40% in each survey year, which is in line with former studies that have reported prevalence rates between 40% and 50% in the oldest old people (Corrada et al., 2008; Doblhammer & Barth, 2018). The evidence indicates decreasing prevalence (Harrison et al., 2020; Wu et al., 2016) and incidence rates for dementia among younger old people in Western countries (Gao et al., 2019; Sullivan et al., 2019), but we are not aware of any such results for the oldest age groups. Our study suggests that the prevalence of dementia remains high among very old people, even though it may be slightly declining. Factors underlying this decline likely include reduced cardiovascular risk factors and a rising level of education (Satizabal et al., 2016). A recent study among 70-year-old Finnish people found a decline in the proportion of people with cardiovascular risk factors for dementia, improved educational level, and better performance in cognitive tests, suggesting a positive development in subsequent cohorts regarding the risk of dementia (Vire et al., 2020). Yet, as age is by far the strongest risk factor for dementia, the impact of these changes on the oldest old is difficult to predict. However, since the oldest old is the fastest growing population segment in Finland, as it is in several other countries worldwide, the absolute number of people having dementia is increasing and will most likely continue to increase in the future.

It is not straightforward to compare our findings with earlier results because most studies also include younger people and the types and number of morbidities included in these studies vary. In addition, most studies are conducted either in care settings or exclude long-term care residents, while our research is population-based and includes long-term care residents. In our study, the participants with dementia had more chronic conditions than participants without dementia in most years, and the number of conditions in this group increased over the study period (2001–2018). This is in line with previous research which shows an increasing prevalence of major age-related chronic conditions in the general older population (Christensen et al., 2009; Crimmins et al., 2019). Also, a recent register-based nationwide study on comorbidity trends during the last years of life in Finnish dementia patients aged 70 years and over, found an increasing burden of comorbidities from 2001 to 2013 (Vargese et al., 2021). The number of comorbidities is highly dependent on the range of conditions included in the analysis, and therefore it is understandable that some earlier studies have found more comorbid chronic conditions with dementia than we did (Clague et al., 2016; Nelis et al., 2018).

The participants in our study were exceptionally old; therefore, the findings cannot be generalized to younger age groups with dementia. Yet our results showed rather similar comorbidities of dementia as reported in previous studies. Hypertension and osteoarthritis are the most common comorbidities in major age-related conditions such as stroke, diabetes, and dementia (Griffith et al., 2019), and they also occurred frequently in our study. Both conditions were more likely to occur among participants without dementia, but their frequency increased over the study period especially among participants with dementia, as did the frequency of diabetes. Also, hip fracture was in most years more prevalent among participants with dementia, possibly reflecting the lowered functional ability associated with dementia and the increased risk for falls (Lach et al., 2017).

Finland has a universal health care system that largely remained unchanged throughout our study period, although diagnostic and therapeutic practices have improved. Even so, it is possible that particularly the results from the early years of our study reflect underdiagnosis of some chronic conditions with less prominent symptoms such as hypertension, especially among people with advanced dementia (Bauer et al., 2014). It has been pointed out that people with dementia may lack access to care and the quality of their care tends to be poorer (Bunn et al., 2014). In addition, cognitive impairment may lead to underreporting of symptoms (Doraiswamy et al., 2002). Both clinical practice and research are currently paying increasing attention to chronic conditions among the oldest old and those with dementia as well as to the importance of early diagnosis of cognitive problems. This refocus is in response to the sharp rise in the number of the oldest old, increasing life expectancy even at very old age, and a health policy emphasis on supporting the functioning and independence of older individuals. In addition, there is growing evidence of the benefits of active treatment of conditions such as hypertension in older individuals (Beckett et al., 2008). Therefore, it is likely that improved diagnostic practices together with longer survival with chronic disease (Enroth et al., 2020) are major contributing factors behind the increasing prevalence of hypertension and diabetes. It is noteworthy that despite these developments, our findings showed not an increase but rather a slightly decreasing trend in the frequency of dementia.

Most conditions included in this study have been found to be associated with dementia, either as a risk factor (Grande et al., 2021) or as a consequence of dementia. These conditions include stroke, Parkinson’s disease, and depression, which are more likely to occur in people with dementia than in those without dementia (Bauer et al., 2014; Clague et al., 2016; Sherzai et al., 2016). Disabling conditions, such as stroke, Parkinson’s disease, depression, and hip fracture were also in our study more prevalent among people with dementia. Depression showed high odds to occur with dementia throughout the study period, whereas the association of stroke, Parkinson’s disease, and hip fracture with dementia varied. Diabetes has been identified as a risk factor for dementia (Rastas et al., 2010), but in our study, its prevalence was not higher among participants with dementia. The role of hypertension in the onset of dementia among the oldest old is controversial even if there is strong evidence of its significance in midlife (Ou et al., 2020). In one study, late-onset hypertension (over 80 years of age) has been associated with a lower risk for dementia in people aged over 90 (Corrada et al., 2017), yet the causal associations are difficult to establish, as the pathological changes develop for several years, even decades, before the diagnosis. As long-living individuals are known to be healthier at younger old age than their age peers who die earlier (Doblhammer & Barth, 2018), it is likely that most of our study population have survived to a rather old age free from dementia or other major conditions, and their comorbidity profile may to some extent differ from that of younger old people.

We found a rather high prevalence of depression (over 24%) in people with dementia in every study year. This is at the same level (Lyketsos et al., 2002; Savva et al., 2009) or higher (Sherzai et al., 2016) than the figures reported for people aged over 65 years. Depression is one of the conditions often associated with dementia, since depression earlier in life has been found to be a risk factor for dementia, and depression can also be a prodrome for dementia (Enache et al., 2011; Grande et al., 2021). In line with Sherzai et al. (2016), we found that depression was more common among participants with dementia than those without, but it showed a tendency to decrease over time, as also indicated by a recent Finnish register-based study (Vargese et al., 2021). Severe clinical depression is quite rare in very old age, but the prevalence of depressive disorders is known to be relatively high (Luppa et al., 2012). The wording of the item in the Vitality 90+ survey (depression, depressed mood) likely has contributed to the rather high prevalence of depression seen in this study.

Our study consisted of six identical cross-sectional surveys spanning a long, 17-year timeline which enabled us to study trends during a reasonably long period. The study population was exceptionally old, and the exhaustive population registers available in Finland allowed us to include all inhabitants in a defined area, irrespective of their place of residence or health status. Excluding long-term care residents and people with poor health from surveys is known to result in underestimated prevalence rates for chronic conditions in older populations (Kelfve et al., 2013). The decision to allow proxy responses meant we also obtained information from participants who would not have been able to answer themselves. As expected, proxy responses were more common among those living in long-term care and having dementia. The response rate was high in every survey year. Even though the range of chronic conditions included was limited, all the most significant and most common diseases were covered. Given the high response rate and the inclusion of long-term care residents and proxy respondents, it is reasonable to assume that our study also includes severe cases of dementia. Since we did not have information on the onset of dementia or comorbidities, we were not in the position to draw conclusions about the stage of dementia and its consequences. Also, the study focused solely on population-based time trends using independent cross-sections and did not follow the incidence or change in morbidity at the individual level.

We had access to dates of death for the total basic population and hence were able to compare mortality between respondents and non-respondents two, three, and 4 months after each Vitality 90+ survey. In each survey round, mortality was higher for non-respondents than for respondents (data not shown). Therefore, the prevalence rate reported for dementia and other chronic conditions is most likely an underestimate since those who did not answer the survey were probably in poorer health than the respondents. The response rate, although high in every round, was slightly lower in 2018. However, the mortality rate after the survey in 2018 was similar to that in previous survey years, suggesting no greater mortality selection in 2018. Therefore, in our understanding, the findings are comparable across the study years.

The data for this study was based on self-reports, the method of choice in most surveys estimating the prevalence of chronic conditions in older populations (Christensen et al., 2009). Self-reports are the most feasible and often the only available method for collecting representative population-based information. It is often assumed that self-reports underestimate the prevalence of chronic conditions, but Christensen et al. (2009) suggest that certain conditions are in fact overreported. The increasing trend in prevalence rates for most chronic conditions is seen in both self-reported data and medical records (Christensen et al., 2009).

We are aware that cognitive decline may undermine the reliability of information collected from the oldest old people, and we have done our utmost to evaluate and limit the extent of this potential problem. First, it should be noted that the majority of our sample did not have cognitive decline, which is consistent with earlier studies in this age group (Corrada et al., 2008; Doblhammer & Barth, 2018). Prior research also suggests that people with mild to moderate cognitive decline are able to assess their health status (Walker et al., 2004) and most of those with diagnosed dementia do report their memory problems (Campbell et al., 2008). Second, for participants with more severe dementia, information was received from family members or care staff. In addition to the 30–44% of proxy respondents for participants with dementia, 16–30% received help from others in answering the survey (data not shown). Thus, less than half of the participants with dementia gave the information entirely independently. Third, in an earlier round of the Vitality 90+ survey, we have compared self-reports of chronic conditions with medical records (Goebeler et al., 2007). We found that participants had a tendency to overreport rather than underreport dementia, depression, and osteoarthritis. Agreement between the survey and medical records was greatest for Parkinson’s disease, hip fracture, and diabetes. Inter-source agreement on chronic conditions was not dependent on cognitive decline recorded by a physician. Compared to studies on self-reported morbidity in younger old people, the disagreement followed the same pattern but was larger in the Vitality 90+ sample. In all, we certainly recognize the uncertainties in the self-reported information collected among the oldest old, but on the balance of evidence we believe that our data is sufficiently reliable and can usefully contribute to our understanding of the health and morbidity of this age group.

As for our results on time trends, possible sources of uncertainty from self-reported information in a trend study include changes in diagnostic practices and reporting behavior, and respondents’ awareness of the conditions queried (Galenkamp et al., 2014). However, these challenges apply to every study conducted over extended period of time and are effectively beyond the control of research teams.

The results of this study contribute to our understanding of the health of the rapidly growing oldest old population, which has an exceptional morbidity profile. As dementia is most often accompanied by other chronic conditions, new clinical phenotypes may occur. Current clinical guidelines usually focus on single diseases, and where guidelines for multimorbidity do exist (Boyd et al, 2019), they do not take into account the specific characteristics of dementia. Comorbidities affect the lives of people with dementia and their caregivers (Bunn et al., 2014) since they are associated with problems in self-care and mobility and with lower quality of life (Doraiswamy et al., 2002; Nelis et al., 2018). Hence, comorbidities aggravate the negative effects of dementia. The increasing number of people with dementia and the increasing comorbidity burden pose new challenges not only for clinical guidelines and practice, but also for research.

## Conclusions

Dementia remains a highly prevalent condition among the oldest old people and it is associated with an increasing comorbidity burden. This presents a significant challenge for the people living with dementia, their caregivers, and the care system. Our results show an increasing prevalence of hypertension, diabetes, and osteoarthritis which likely reflects improved diagnostics and treatment of these conditions in general and especially among individuals with dementia, and longer survivorship with these conditions. Depression, even though it is showing a slightly declining trend, is notably more common among individuals suffering from dementia than others. Yet it is possible that some conditions, particularly those with fewer symptoms, are still underdiagnosed in people with dementia. This is a question that warrants closer investigation. This study implies that increasing longevity likely leads to an increasing number of chronic conditions comorbid with dementia and a growing prevalence of multimorbidity. It is therefore crucial that further epidemiological, clinical, and social research is conducted on dementia comorbidity among the most rapidly growing population group, the oldest old.

## Supplemental Material

Supplemental Material - Dementia and Related Comorbidities in the Population Aged 90 and Over in the Vitality 90+ Study, Finland: Patterns and Trends From 2001 to 2018 Click here for additional data file. Supplemental Material for Dementia and Related Comorbidities in the Population Aged 90 and Over in the Vitality 90+ Study, Finland: Patterns and Trends From 2001 to 2018 by Pauliina Halonen, Linda Enroth, Esa Jämsen, Saritha Vargese, and Marja Jylhä in Journal of Aging and Health
