# Supplementary material for: Dementia and Related Comorbidities in the Population Aged 90 and Over in the Vitality 90+ Study, Finland: Patterns and Trends From 2001 to 2018
Source: J Aging Health. 2022 Oct 18;35(5-6):370–82. doi: 10.1177/08982643221123451 (PMC10150268; doi:10.1177/08982643221123451)
Supplement: Supplemental Material - Dementia and Related Comorbidities in the Population Aged 90 and Over in the Vitality 90+ Study, Finland: Patterns and Trends From 2001 to 2018 [file sj-pdf-1-jah-10.1177_08982643221123451.pdf]

Supplement Table 1. Prevalence of disease combinations among participants with dementia in each study year

	2001	2003	2007	2010	2014	2018
	% (*n*)	% (*n*)	% (*n*)	% (*n*)	% (*n*)	% (*n*)
Hypertension & heart disease	21.6 (81)	26.3 (115)	22.1 (97)	25.0 (135)	32 (223)	37.6 (289)
Hypertension & diabetes	5.6 (21)	5.5 (24)	5.7 (25)	6.3 (34)	10.5 (73)	15.1 (116)
Hypertension & osteoarthritis	10.4 (39)	11.2 (49)	14.6 (64)	18.3 (99)	24.2 (169)	27.2 (209)
Hypertension & hip fracture	4.8 (18)	7.1 (31)	5.5 (24)	8.0 (43)	10.3 (72)	10.3 (79)
Hypertension & depression	11.2 (42)	12.8 (56)	10.5 (46)	13.9 (75)	13.8 (96)	17.8 (137)
Heart diasease & cancer	7.7 (29)	7.3 (32)	6.4 (28)	-	9.3 (65)	8.3 (64)
Heart disease & diabetes	9.6 (36)	14.4 (32)	6.4 (28)	7.6 (41)	8.7 (61)	11.7 (90)
Heart disease & osteoarthritis	20.8 (78)	21.5 (94)	16.4 (72)	22.9 (124)	24.2 (169)	25.4 (195)
Heart disease & hip fracture	9.1 (34)	11.2 (49)	7.1 (31)	11.3 (61)	10.7 (75)	9.6 (74)
Heart disease & depression	18.9 (71)	19.9 (87)	14.1 (62)	16.5 (89)	14.3 (100)	14.8 (114)
Cancer & osteoarthritis	4.5 (17)	4.1 (18)	3.9 (17)	-	6.9 (48)	6.5 (50)
Osteoarthritis & hip fracture	5.9 (22)	8.7 (38)	4.3 (19)	8.7 (47)	10.0 (70)	8.2 (63)
Osteoarthritis & depression	11.5 (43)	13.5 (59)	10.5 (46)	13.7 (74)	13.6 (95)	13.3 (102)
Hypertension, heart disease & osteoarthritis	7.2 (27)	9.6 (42)	8.2 (36)	11.8 (64)	15.9 (111)	18.8 (144)
Hypertension, heart disease & depression	7.7 (29)	9.6 (42)	6.4 (28)	8.7 (47)	8.3 (58)	11.7 (90)
Hypertension, osteoarthritis & depression	4.5 (17)	5.7 (25)	5.2 (23)	7.0 (38)	8.3 (58)	9.4 (72)
Heart disease, osteoarthritis & depression	6.7 (25)	9.1 (40)	5.7 (25)	9.6 (52)	8.2 (57)	8.1 (62)

Supplement Table 2. Association of combinations of chronic conditions with dementia in the six study waves. Logistic regression analysis with odds ratios (OR) and 95% Confidence Intervals (CI) adjusted for age and gender. Odds >1.00 indicate higher prevalence among people with dementia

	2001		2003		2007		2010		2014		2018	
	**OR**	**95% CI**	**OR**	**95% CI**	**OR**	**95% CI**	**OR**	**95% CI**	**OR**	**95% CI**	**OR**	**95% CI**
Hypertension & heart disease	1.18	0.85-1.65	1.17	0.86-1.59	**0.62**	**0.46-0.83**	**0.60**	**0.47-0.77**	**0.78**	**0.63-0.96**	1.00	0.83-1.21
Hypertension & diabetes	**2.25**	**1.11-4.55**	1.48	0.80-2.73	0.72	0.43-1.22	**0.67**	**0.44-1.03**	1.05	0.76-1.45	1.11	0.86-1.45
Hypertension & osteoarthritis	0.78	0.51-1.19	**0.64**	**0.44-0.94**	**0.44**	**0.32-0.62**	**0.54**	**0.41-0.71**	**0.67**	**0.53-0.84**	**0.74**	**0.60-0.91**
Hypertension & hip fracture	0.98	0.52-1.84	1.00	0.60-1.66	**0.45**	**0.27-0.74**	0.80	0.54-1.20	1.05	0.75-1.46	1.33	0.96-1.84
Hypertension & depression	**2.49**	**1.48-4.20**	**2.10**	**1.32-3.32**	1.30	0.84-2.03	**2.04**	**1.40-2.97**	**1.99**	**1.43-2.76**	**3.26**	**2.39-4.44**
Heart disease & cancer	1.65	0.94-2.90	1.20	0.71-2.02	0.90	0.54-1.52	-	-	0.96	0.68-1.35	0.78	0.56-1.08
Heart disease & diabetes	**1.79**	**1.07-3.00**	1.17	0.69-1.99	0.96	0.57-1.63	1.00	0.65-1.52	1.05	0.74-1.49	1.26	0.93-1.69
Heart disease & osteoarthritis	0.91	0.65-1.26	1.01	0.73-1.38	**0.49**	**0.36-0.68**	**0.76**	**0.58-0.98**	0.84	0.67-1.06	0.94	0.76-1.17
Heart disease & hip fracture	1.02	0.63-1.64	1.25	0.81-1.92	**0.54**	**0.34-0.85**	1.17	0.81-1.70	1.18	0.85-1.65	1.34	0.96-1.88
Heart disease & depression	**2.3**	**1.55-3.43**	**2.39**	**1.63-3.52**	1.31	0.89-1.94	**2.76**	**1.89-4.02**	**2.32**	**1.67-3.24**	**2.94**	**2.12-4.08**
Cancer & osteoarthritis	1.09	0.57-2.11	0.80	0.43-1.49	0.79	0.42-1.48	-	-	1.01	0.68-1.49	**0.59**	**0.41-0.83**
Osteoarthritis & hip fracture	0.84	0.48-1.47	1.36	0.83-2.23	**0.32**	**0.19-0.54**	0.82	0.55-1.22	1.30	0.92-1.85	1.03	0.73-1.45
Osteoarthritis & depression	1.24	0.80-1.92	**2.02**	**1.30-3.15**	1.10	0.72-1.69	**2.11**	**1.44-3.09**	**1.94**	**1.40-2.69**	**2.7**	**1.92-3.79**
Hypertension, heart disease & osteoarthritis	0.91	0.54-1.51	0.92	0.60-1.42	**0.41**	**0.27-0.62**	**0.57**	**0.41-0.79**	0.77	0.59-1.00	0.95	0.75-1.20
Hypertension, heart disease & depression	**2.41**	**1.30-4.46**	**2.59**	**1.48-4.55**	0.98	0.58-1.66	**2.16**	**1.34-3.48**	**2.06**	**1.36-3.12**	**3.21**	**2.20-4.68**
Hypertension, osteoarthritis & depression	1.51	0.74-3.08	1.77	0.92-3.38	0.88	0.50-1.53	**1.73**	**1.05-2.84**	**1.76**	**1.18-2.64**	**2.82**	**1.88-4.25**
Heart disease, osteoarthritis & depression	1.15	0.66-2.01	**2.15**	**1.25-3.70**	0.83	0.49-1.42	**2.82**	**1.72-4.60**	**1.66**	**1.12-2.48**	**2.53**	**1.66-3.87**

Notes: Analyses were conducted for each combination separately.
Bolding indicates statistically significant association.

Supplement Table 3. Prevalence of disease combinations among participants without dementia in each study year

	2001	2003	2007	2010	2014	2018
	% (*n*)	% (*n*)	% (*n*)	% (*n*)	% (*n*)	% (*n*)
Hypertension & heart disease	18.8 (94)	23.3 (116)	31.4 (155)	36.3 (262)	37.6 (347)	37.4 (407)
Hypertension & diabetes	2.6 (13)	4.0 (20)	7.9 (39)	10 (72)	10.5 (97)	14.2 (154)
Hypertension & osteoarthritis	12.8 (64)	16.6 (83)	28.2 (139)	29.2 (211)	32.3 (298)	32.8 (357)
Hypertension & hip fracture	4.8 (24)	6.8 (34)	10.8 (53)	9.4 (68)	9.6 (89)	7.8 (85)
Hypertension & depression	4.8 (24)	6.4 (32)	8.7 (43)	7.3 (53)	7.6 (70)	6.3 (68)
Heart diasease & cancer	4.8 (24)	6.0 (30)	6.9 (34)	-	9.8 (90)	10.3 (112)
Heart disease & diabetes	5.6 (28)	12.6 (33)	6.5 (32)	8.2 (59)	8.9 (82)	9.7 (106)
Heart disease & osteoarthritis	22.2 (111)	21.6 (108)	28.4 (140)	28.1 (203)	27.5 (254)	25.7 (280)
Heart disease & hip fracture	8.6 (43)	9.0 (45)	11.8 (58)	9.3 (67)	9.1 (84)	7.1 (77)
Heart disease & depression	9.4 (47)	9.4 (47)	11.6 (57)	6.7 (48)	7.0 (65)	5.6 (61)
Cancer & osteoarthritis	4.2 (21)	5.2 (26)	5.1 (25)	-	6.9 (64)	10.3 (112)
Osteoarthritis & hip fracture	6.8 (34)	6.4 (32)	12.4 (61)	9.7 (70)	7.6 (70)	7.6 (83)
Osteoarthritis & depression	9.4 (47)	7 (35)	10.1 (50)	6.9 (50)	7.9 (73)	5.3 (58)
Hypertension, heart disease & osteoarthritis	7.8 (39)	10.4 (52)	18.1 (89)	18.8 (136)	19.6 (181)	19.0 (207)
Hypertension, heart disease & depression	3.4 (17)	3.8 (19)	6.7 (33)	4.2 (30)	4.4 (41)	4.0 (43)
Hypertension, osteoarthritis & depression	3 (15)	3.2 (16)	6.3 (31)	4.2 (30)	5.1 (47)	3.5 (38)
Heart disease, osteoarthritis & depression	5.8 (29)	4.4 (22)	7.1 (35)	3.6 (26)	5.4 (50)	3.3 (36)
